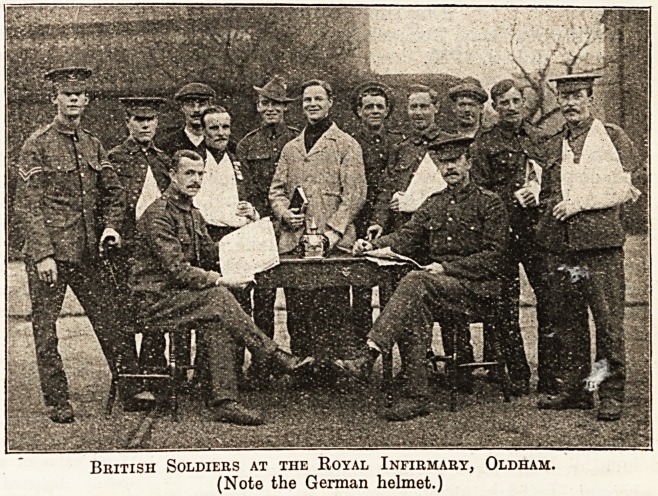# Oldham's Work for the Wounded

**Published:** 1915-07-17

**Authors:** 


### OLDHAM'S WORK FOR THE WOUNDED.

(FROM A CORRESPONDENT.)

Until January the forty beds placed at the disposal of the military authorities last October by the Oldham Royal Infirmary were occupied by wounded Belgian soldiers. It has since, however, been found necessary to reduce this number to twenty, as there were so many civilian patients awaiting admission.

Belgian Soldiers at the Royal Infirmary, Oldham.
(Looking *at* [*sic*] a war map.)

The soldiers' ward is bright and cheerful, and the men are quite contented, in spite of the fact that many of them have been there for some time. They have the inevitable gramophone — the records of which show signs of hard usage—various games, and always cigarettes. Those who can, play football; the ball has been mended with sticking-plaster, but that does not seem to mar their enjoyment.

The men are sent to Oldham from the military hospital in Manchester; when well enough to leave, those who do not go home on furlough are sent to a Red Cross convalescent home in Greenfield. Soldiers from many parts of the world are there; at the present time there is a Canadian, an Australian, one from South Africa, one from Fiji, one from an Indian cavalry regiment, and many others.

Several of the Belgians unfit for further military service have remained in Oldham, and found employment in various ironworks, earning about 35s. per week.

One wonders why the soldier seems more child-like than the rest of us. Perhaps it is not so, but only that we are very interested in him, and note his every action, his sense of property, the pleasure he shows in a small gift, and the way in which he guards his treasures, taking us back to the days when we carried our most treasured possession to bed with us, lest some calamity should befall it in the night. There is one man in Oldham who always takes a German bayonet with him to the bathroom, so carefully does he prize it.

There are two Voluntary Aid Detachment hospitals. Woodfield, given by Mrs. C. E. Lees, the late Mayor, is an auxiliary military hospital. There are fifty beds. A lady doctor is acting as matron, and there are three hospital-trained sisters, assisted by Red Cross nurses. A nursing home has also been lent to the soldiers.

Men from many battles have been nursed in the infirmary—from Dixmude, Ypres, La Bassée, Hill 124, etc. Sister talks to them, and they tell her tales, some of them amusing and some very pitiful. A number of the nursing staff are abroad, nursing in Havre, in Alexandria, under canvas, and on hospital ships.

British Soldiers at the Royal Infirmary, Oldham.
(Note the German helmet.)

Altogether those of the staff of the Oldham Royal Infirmary who have remained at the institution have already found virtue to be its own reward in the practical shape of much hard and interesting work, brightened by many happy and touching memories.

## Figures and Tables

**Figure f1:**
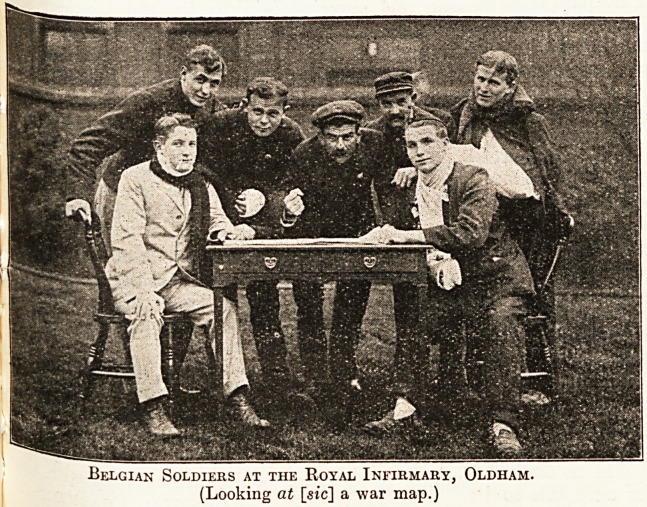


**Figure f2:**